# Effect of Process Factors on Tensile Shear Load Using the Definitive Screening Design in Friction Stir Lap Welding of Aluminum–Steel with a Pipe Shape

**DOI:** 10.3390/ma14195787

**Published:** 2021-10-03

**Authors:** Leejon Choy, Seungkyung Kim, Jeonghun Park, Myungchang Kang, Dongwon Jung

**Affiliations:** 1 Graduate School of Convergence Science, Pusan National University, Busan 46241, Korea; leejonee@hanmail.net (L.C.); kskyeong9970@gmail.com (S.K.); jeonghun.park@ctr.co.kr (J.P.); 2 Faculty of Mechanical, Jeju National University, Jeju-si 63243, Korea

**Keywords:** friction stir lap welding, definitive screening design (DSD), tensile shear load, tool penetration depth, plunge depth

## Abstract

Recently, friction stir welding of dissimilar materials has emerged as one of the most significant issues in lightweight, eco-friendly bonding technology. In this study, we welded the torsion beam shaft—an automobile chassis component—with cast aluminum to lighten it. The study rapidly and economically investigated the effects of friction stir welding and process parameters for A357 cast aluminum and FB590 high-strength steel; 14 decomposition experiments were conducted using a definitive screening design that could simultaneously determine the effects of multiple factors. Friction stir welding experiments were conducted using an optical microscope to investigate the tensile shear load behavior in the welding zone. In addition to understanding the interactions between tool penetration depth and plunge speed and tool penetration depth and dwell time, we investigated and found that tool penetration depth positively affected the size of the hooking area and contributed to the stabilization and size reduction of the cavity. The experimental results showed that the plunge depth and tool penetration depth effects were most important; in this case, the plunge depth negatively affected the magnitude of tensile shear load, whereas the tool penetration depth had a positive effect. Therefore, when selecting a tool, it is important to consider the plunge depth and tool penetration depth in lap welding.

## 1. Introduction

Recently, environmental protection and energy-saving strategies—including weight reduction and miniaturization—have emerged as critical issues in the automobile and other industries [1,2,3]. Reducing a vehicle’s weight improves its overall performance and fuel economy; therefore, replacing steel with Al alloys is a possible alternative. However, the complete replacement of steel with Al alloys has technical limitations, such as the fact that Al alloys do not have finite fatigue limits. The high strength, excellent creep resistance, and formability of steel and the low density, high thermal conductivity, and excellent corrosion resistance of aluminum alloys can be combined into one hybrid structure. There are four main aspects of the bonding difficulty [4]. The current welding methods—such as low energy input fusion welding and brazing—can induce the formation of an intermetallic compound (IMC) layer with a certain thickness. The evolution of brittle IMCs, which are generated during the interfacial reaction between solid steel and liquid aluminum, can significantly influence the mechanical properties of Al/steel joints. Therefore, new methods must be developed to realize the rapid development of dissimilar aluminum alloy and steel welding [5]. Invented and patented in 1991 by The Welding Institute in Cambridge, UK, friction stir welding (FSW) is an energy-efficient, environmentally friendly, and versatile bonding technology [6]. FSW is a solid-state bonding method that has low thermal deformation and high bonding efficiency in joining dissimilar materials such as aluminum and steel. Because of its eco-friendliness and economic benefits, it is actively being introduced to the manufacture of automobile parts. A coupled torsion beam axle (CTBA) is installed at the rear end of a vehicle, connecting the tire to the body. This component absorbs vibrations or shocks on the road surface to improve ride comfort, supports braking and lateral forces received from tires, and adjusts the roll angle of the vehicle when cornering with torsional stiffness of the body [7]. In automobiles and other areas, the two materials must be combined efficiently to replace steel with Al alloys. Park et al. [8] investigated and found that proper mixing of materials occurred in the nugget region when the stronger base material was placed on the advancing side in the butt joint of Al and Steel. As an applied study of dissimilar Al and steel FSW, Park et al. [9] performed FSW between a 3 mm thick A357 cast Al flat plate and FB590 high-strength steel flat plate and reported achieving 72.8% strength compared to the Al base material. A characteristic feature of lap FSW (FSLW) is the geometric defect called a “hook” that occurs at the interface of two weld sheets. FSW is a complex thermomechanical process; the final performance—including mechanical strength—of FSW joints is closely related to the tool geometry and welding parameters.

To reduce defects and increase the mechanical strength of these FSW joints, many researchers have conducted studies on tool shape and process parameters [10,11,12,13,14,15,16,17,18,19]. Research has also been conducted on the importance of the tool shoulder [20,21,22,23,24,25,26]. Trimble et al. [20] considered the effect of tool shape and rotational speed on the AA2024-T3 flat plate material. The results indicated that it is possible to achieve good weld quality at speeds up to 355 mm/min by welding with a scroll shoulder and triflute pin at a rotational speed of 450 rpm. The recently developed “scroll”-shaped tool shoulder is particularly desirable for curved joints. Trueba Jr. et al. [21] reported that the FSW tool shoulder with a raised helical design produces the best welds in terms of surface quality and mechanical properties in butt-welded aluminum 6061-T6 plates using six differently designed tools of Ti-6Al-4V material. Research has been conducted on process-parameter-related tools to reduce defects and increase the mechanical strength of FSW joints [27,28]. The normal force must be varied in the following four stages: tool plunge stage, dwell stage, welding stage, and tool retreating stage. Process parameters affect the normal force and temperature in FSW processes. In addition to tools, research has been conducted on process parameters to reduce defects and increase the mechanical strength of FSW joints [29,30,31,32,33,34]. Baskoro et al. [29] conducted experiments and analyses for plunge speeds of 2, 3, and 4 mm/min and dwell times of 0, 2, and 4 s in the high-speed micro-friction stir spot welding (µFSSW) of Al A1100 with a thickness of 0.4 mm and revealed that a dwell time of 2 s was the most important welding parameter in the µFSSW process. Li et al. [30] investigated the correlation between microstructure and mechanical properties by performing FSSW at a plunge depth of 0.1 mm with a dwell time between 1 and 9 s on dissimilar lap joints of 2 mm thick 1060 aluminum and T2 copper plates. At shorter dwell times, a discontinuous layer of CuAl_2_ forms and mixes with the CuAl formed at the interface due to insufficient heat input. Longer dwell times can lead to higher peak temperatures but lower plunging forces and torques. They are characterized by the formation of a continuous CuAl_2_-CuAl-Al_4_Cu_9_ stacked layer at the interface, resulting in microcracks. They also reported the need for an appropriate dwell time. Zheng et al. [31] reported that the pin failed at low loads that did not reach the nickel alloy surface; in contrast, the maximum tensile shear strength of the lap joint was obtained at a plunge depth of 0.3 mm and reached 7.9 kN. The plunge depth of the joint was determined to have a significant effect on the strength of FSW lap joints of Al alloy (2A70) and nickel-based alloys through experiments at various plunge depths from 0 to 0.5 mm. Devanathan et al. [32] investigated the effect of plunge depths of 0 and 0.2 mm and reported that increasing the plunge depth reduced mechanical performance, leading to defective welds in 6063Al butt welding; their study used a single tool with a shoulder diameter of 24 mm, pin diameter of 4 mm, and pin length of 5 mm. Wei et al. [33] performed FSLW of a 3 mm thick Al (1060Al) sheet and a 1 mm thick stainless steel (SUS321, austenite) sheet using a 6 mm diameter pin, with an insertion depth of 2.8 and 3.2 mm, and found that the greater the tool penetration depth, the greater the tensile strength. Regensburg et al. [34] investigated hooking and IMC formation with the pin lengths at the junctions being 1.8, 2.0, 2.2, 2.4, and 2.6 mm at the lap joint of EN AW1050/CW024A material with a thickness of 2 mm. It was found that plunging into the lower copper sheet of about 0.2 mm by a pin length of 2.2 mm yielded the highest breaking load. The further increase in pin length led to the formation of hooking defects, which resulted in void formation at the interface and failure within the thin aluminum sheet area. However, the effect of plunge depth and tool penetration depth could not be considered simultaneously. FSLW is more difficult than butt FSW. Many studies have been conducted on FSLW between Al alloys [11,13,28,34]. Significant research has been conducted on FSLW of Al–Mg [12], Al–Cu [30,35], Al–Ni [31], etc.; however, in practical cases, many studies have been conducted on FSLW of Al alloy and steel [5,9,33,36,37,38,39]. Choy et al. [37] performed FSW of 3 mm thick A357 cast Al pipes and FB590 high-strength steel to investigate the significance of the influence of process parameters. As a result, the plunge depth effect was most dominant. However, it was not possible to isolate the effects of plunge depth and tool penetration depth using a single tool. Because FSLW is a different structure from butt FSW, geometrical defects occur [11,34,39,40]. FSW of pipes is tedious owing to its complex geometry, and therefore, research papers are rare. The butt FSW between Al alloy pipe [41,42,43,44,45,46] and butt FSW between steel pipes are mainly performed [47,48]. Choy et al. [37] performed FSLW of 3 mm thick A357 cast Al and FB590 high-strength steel pipes. Two-factor analysis has been used to identify the factors that affect characteristic values using the design of experiment (DOE) method [49,50].

In previous research, although it was possible to conduct experiments on some of the individual factors, it was difficult to conduct experiments on the relative importance of multiple process factors, owing to time and cost. However, DSD is an innovative experimental method that can reduce time and cost, enabling researchers to select and test the relative importance of multiple process factors. Choy et al. [37] reported the effect of each process factor on tensile shear load (TSL) by performing FSW based on the DSD experimental design for five process factors with three levels for the lap joint of each 3 mm thick pipe-shaped A357 cast Al and FB590 high-strength steel, using a tool of a single dimension. However, the change in the plunge depth affects the tool penetration depth owing to the experiment with a single dimension tool pin; hence, the exact effect of the plunge depth cannot be obtained. Most studies to date have attempted to establish correlations between post-welding properties and the main FSW process variables, welding speed, and tool rotation speed, and one or two individual variables. Additionally, studies on the correlation of multiple process variables are limited.

Therefore, this study considers the influences on the TSL at the time of dissimilar FSLW bonding of pipe-shaped A357 cast Al and FB590 high-strength steel. To isolate the mutual effect of plunge depth and tool penetration depth, which were not considered at all in previous papers, four types of tools with different pin lengths according to the number of levels were selected and tested accordingly. To investigate the relative importance of a number of process factors, which were rarely addressed in previous papers, the DOE method of DSD was adopted to examine the selection of the multiple process factors for TSL, their relative importance, and the effects of linear and curved relationships. Minitab (Ver. 19, Minitab Ltd., State College, PA, USA) was used for DSD design. In addition, the characteristics of the microstructure and TSL were evaluated according to factor 6 and level 3 (depth of plunge was level 2 [51]).

## 2. Experimental Preparation and Design Methods

### 2.1. Materials and Tools

**Figure 1:**
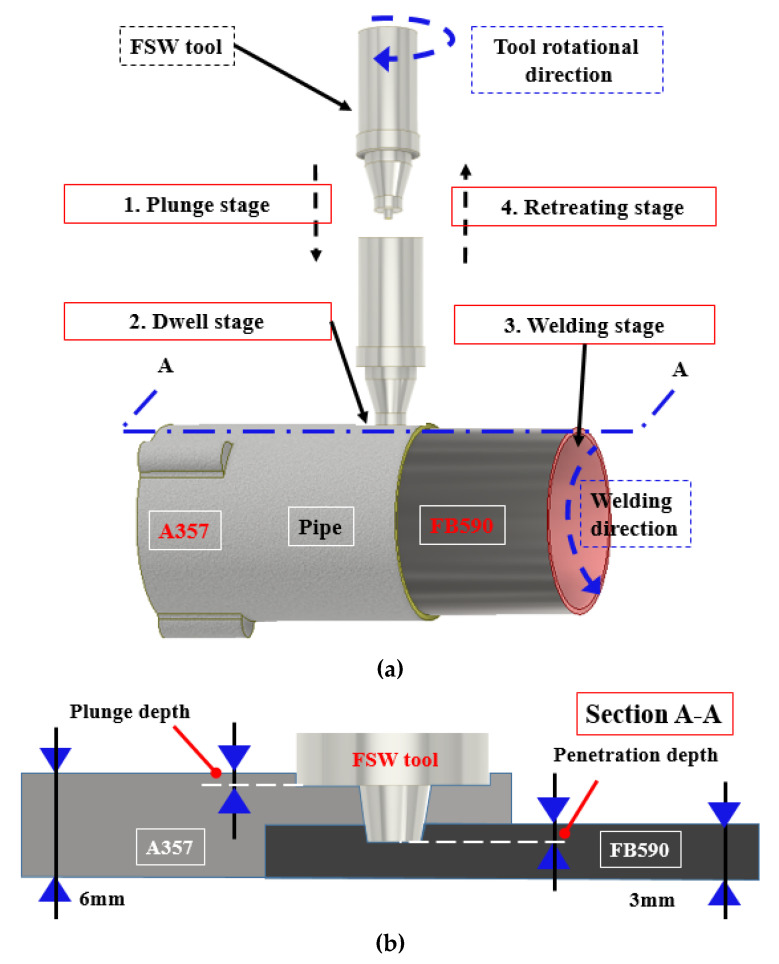
Schematic of FSW experimental configuration. (**a**) Four stages of FSW process; (**b**) plunge depth and penetration depth of FSLW.

The pipes used in this experiment were A357 cast Al and FB590 high-strength steel. The chemical composition of each material is shown in Table 1 [9]. The test specimen, A357 cast Al pipe, was manufactured to have an outer diameter of 111 mm, length of 155 mm, joint thickness of 3 mm, and non-joint thickness of 6 mm. A357 cast Al pipe is subjected to T6 heat treatment after casting and surface treatment through shot peening. FB590 is a high-strength steel pipe with an outer diameter of 105 mm, length of 110 mm, and thickness of 3 mm. Before welding, the aluminum oxide layer of the aluminum alloy was removed with a brush and sandpaper. The two materials were joined by FSLW, as illustrated in Figure 1. Figure 1a shows the four stages of the FSW process, and Figure 1b shows the plunge depth and tool penetration depth of FSLW. The FSW process is primarily classified into four stages: the plunge stage, where the tool descends to the depth of the workpiece; the dwell stage, where the tool stays to provide a constant temperature; the welding stage to join the workpiece; and the retreating stage, where the tool exits after welding is finished.

During the FSW process, heat is generated by friction between the tool and the workpiece, which causes plastic deformation of the workpiece [52]. While the heat softens the material in the shear layer around the tool, the plastic material flow in the shear layer produces localized viscous dissipation heat energy. The combination of the tool rotation and translation leads the softened material to flow from the front of the tool (advancing side (AS)) to the back of the tool (retreating Side (RS)), where it is forged into a joint. In the FSW process, both the heat generation and material flow have crucial effects on the metallurgical characteristics and mechanical properties of the weld joints [53,54]. Furthermore, the preheating effects of the plunge and dwell stages significantly affect the welding force and tool wear [16]. Moreover, the plunge depth and tool penetration depth due to the pin length of the initially selected tool have important effects on welding force and tool wear. Therefore, a complete understanding of both the heat generation and material flow at different stages of the FSW process is imperative in optimizing the process and controlling the microstructures and joint properties.

**Figure 2:**
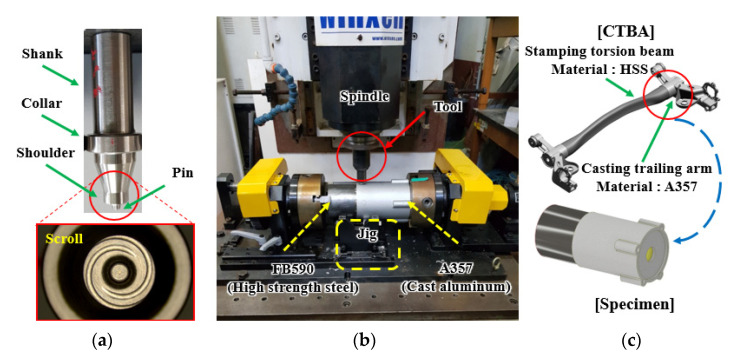
Overall configuration of FSW experiment. (**a**) Photograph of experimental equipment; (**b**) tool and close view of shoulder; (**c**) CTBA and pipe specimen.

The experimental device, shown in Figure 2a, comprises a Winxen milling device that supplies the rotational force of the spindle up to 2000 rpm, a chuck that fixes both sides of FB590 high-strength steel and A 357 cast Al pipe for FSW processing, and a fixing jig consisting of supporting bearings to secure both sides of the FB590 high-strength steel and A357 cast Al pipes for welding. Figure 2b shows the tool used in the FSW processing and the enlarged picture of the scroll shape of the shoulder used to investigate the effect of plunge depth and tool penetration depth. The material and shape dimensions of the tool were selected through a literature review and experiments. The material of the FSW tool was manufactured using W–Ni–Fe alloy, which is a type of heavy alloy. The tool’s pin was processed into a threaded shape with cylindrical tape, and the tool’s shoulder was processed into a parallel scroll shape to increase the *z*-axis vertical force, improving frictional heat and stirring during the FSW joining process.

The shoulder, pin root, and pin diameter of the tool are 10, 5, and 4 mm, respectively. To exclude the correlation between plunge depth and tool penetration depth, four types of tools with a pin length of 2.5, 3, 3.5, and 4 mm were used.

The experiment was conducted by selecting a tool with a pin length according to the plunge depth and tool penetration depth according to the order and levels of the experiment. Figure 2c shows a CTBA, which is the rear wheel suspension of a vehicle, composed of a trailing arm made of casting (cast Al) and a stamping torsion beam made of high-strength steel; also shown is the pipe specimen for the experiment—a part of the trailing arm and a part of the stamping torsion beam are separated.

### 2.2. Definitive Screening Design and Analysis

The DOE table of DSD has 2*m* + 1 runs for *m* factors plus *m* pairs of fold-overs and total centroids. Each run (except all centroids) has a centroid at exactly one factor level, and all other factor levels are designed at the vertices [51,55]. Using the design structure of DSD, a DOE was performed with 14 runs with six factors. Table 2 shows the factors and the number of levels for the DSD.

The following FSW process factors were selected: a tool rotation speed of 1700–1900 rpm, pipe welding speed of 0.1–0.2 rpm, plunge speed of 5–9 mm/min, dwell time of 3–7 s, plunge depth of 0–0.5 mm, and tool penetration depth of 0–1.0 mm. Among the number of levels for the six important factors, only the plunge depth was set to two levels, and all others were set to three levels; the microstructure and TSL characteristics of the joint were evaluated by performing DOE with a standard number of runs of 14 using the DSD method. For structural observation and evaluation of mechanical properties of the joint, tensile test specimens and microstructure observation specimens were prepared through wire processing. The test specimen of TSL was manufactured according to the ASTM E8 standard, and TSL was measured using a tensile tester (AGS-X Shimadzu, Japan). The microstructure observation specimen was polished and then observed through an optical microscope (KH-8700, HIROX, Japan).

## 3. Results and Discussion

### 3.1. Tensile Shear Load Characteristics of Dissimilar Friction Stir Joints

The tools that have an important influence on the FSW characteristics of A357 cast Al and FB 590 high-strength steel pipes were selected through preliminary experiments with reference to the cited papers. Among the process variables that have an important influence—other than tools—six factors were selected as the factors affecting the TSL of dissimilar materials for FSLW, including those not selected by the previous researchers in the experimental plan. Table 3 shows the results of TSL after FSLW of dissimilar materials; in the case of A357 cast Al raw material, the maximum TSL was 7912 N. The highest TSL was 2672.21 N under the conditions of a tool rotational speed of 1800 rpm, welding speed of 0.2 rpm, plunge speed of 5 mm/min, dwell time of 3 s, plunge depth of 0 mm, and tool penetration depth of 0 mm. The TSL value was 897.35 N under the conditions of a tool rotational speed of 1900 rpm, welding speed of 0.2 rpm, plunge speed of 7 mm/min, dwell time of 7 s, plunge depth of 0 mm, and tool penetration depth of 0 mm. In the study by Choy et al. [37], a TSL value of 3500–4500 N could be obtained by using a single tool with a pin length of 3.3 mm and adding the normal force due to the plunge depth.

### 3.2. Definitive Screening Design Model and Main Effect Plot

The model summary in Table 4 shows how well the model explains the observed response variation through process variables with R-Square and R-Square (modified) representing 96.90% and 93.27%. The error (%) of the pre-correction model is 100 (%) − [R-sq] (%) = 3.10 (%) and the error (%) of the modified model is 100 (%) − [R-sq (adj)] (%) = 6.73 (%). The ANOVA table in Table 5 shows the influence of the model terms on the response variable, and statistical significance or influence is judged by the F-statistic and P-value. The appropriate TSL model is a hierarchical model with a small error under the significance level of 10%, and important factors were selected by the DOE method of the DSD step. The influence of the factors on TSL was determined using the coded coefficients and F-statistics and P-values of ANOVA. The regression equation of the TSL value in uncoded units is expressed as Equation (1):

TSL = 1193 + 916 × C − 483.1 × D − 406.1 × E – 3370 × F − 80.0 × C × C + 194.7 × C × F + 512.6 × D × F
(1)

where C is the plunge speed, D is the dwell time, E is the plunge depth, and F is the tool penetration depth.

**Figure 3:**
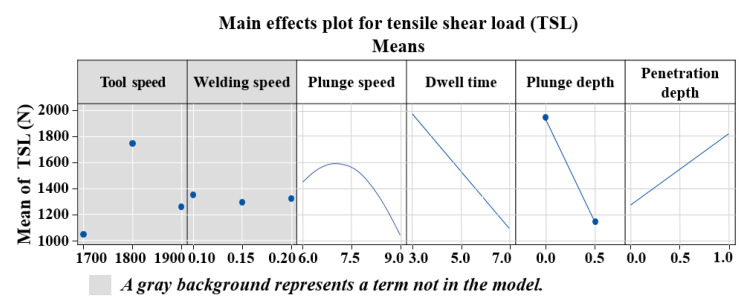
Main effect plots for tensile shear load.

Figure 3 shows the main effect plots for the tensile shear load. This figure shows the average value of tensile shear load according to the number of levels of each of the six factors—tool rotational speed, welding speed, plunge speed, dwell time, plunge depth, and tool penetration depth.

At a given level, it is possible to screen for factors that do not affect TSL with DSD.

Tool rotational speed (A) and welding speed (B) are grayed out in the main effects plot in Figure 3 as they have no effect at any given level. Therefore, the tool rotational speed (A) and welding speed (B) are excluded from the process of finding the maximum value of TSL, and the maximum value of the TSL is found as the remaining four factors that affect it. At a given level, the plunge speed is indicative of the curvature, which is optimal. It was found that dwell time and plunge depth had a negative effect, and only the tool penetration depth had a positive effect. The desired result is high tensile shear load, so the maximum TSL is achieved at a plunge speed of 6.5 mm/min, a dwell time of 3.0 s, a plunge depth of 0.0 mm, and a tool penetration depth of 1.0 mm. This result is inconsistent with the study by Zheng et al. [31], where the plunge depth of dissimilar materials played an important role in the joint tensile strength and increased the maximum breaking load. This does not separate the plunge depth from the tool penetration depth, and the change in the tool penetration depth occurs simultaneously when the plunge depth is changed, resulting in an overall increase in the load when the plunge depth is increased. In addition, Li et al. [30] demonstrated that if the dwell time is short, microcracks occur owing to insufficient heat input, and the hardness may decrease slightly if the dwell time is long; however, the results do not significantly affect the tensile strength. However, this is a result of welding in a steady state, and in the initial stage of welding, the longer the dwell time and the lower the tensile strength. Baskoro et al. [29] presented a result different from this, showing the optimum value at 6.5 mm/min within a given section; their investigation of the effects between process variables showed that the TSL increases as the plunge speed decreases during Al thin plate welding. This is thought to be due to the difference between single materials and dissimilar materials and the difference between butt FSW and FSLW.

### 3.3. Characteristics between the Factors of a Definitive Screening Design

**Figure 4:**
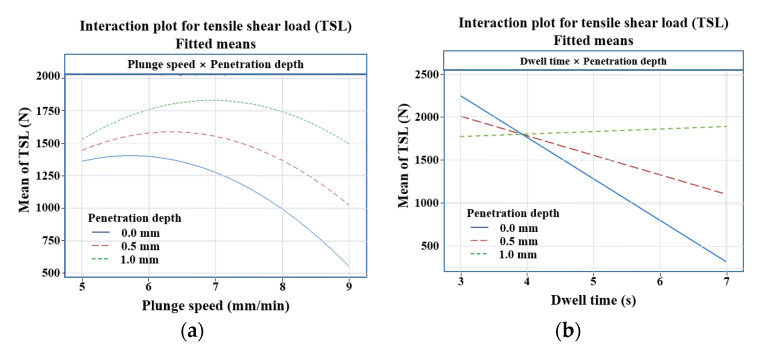
Interaction plot for tensile shear load. (**a**) Penetration depth and plunge speed; (**b**) penetration depth and dwell time.

Figure 4 shows the interaction for TSL. This is according to the interaction between factors in the regression equation of the TSL of Equation (1). Figure 4a is a diagram with the interaction between the plunge speed and the tool penetration depth (C × F) for TSL. In the equation, it is expressed as a curved surface as an effect of the square term of the plunge speed, and it can be seen that an optimal value exists within a given range. It shows the positive effect of increasing TSL with increasing tool penetration depth over the entire range at a given level. Figure 4b is a diagram of the interaction between dwell time and tool penetration depth (D × F) for TSL, where TSL increases with increasing tool penetration depth for dwell times greater than 4 s. On the other hand, when the dwell time is less than 4 s, it shows that the TSL decreases as the tool penetration depth increases.

**Figure 5:**
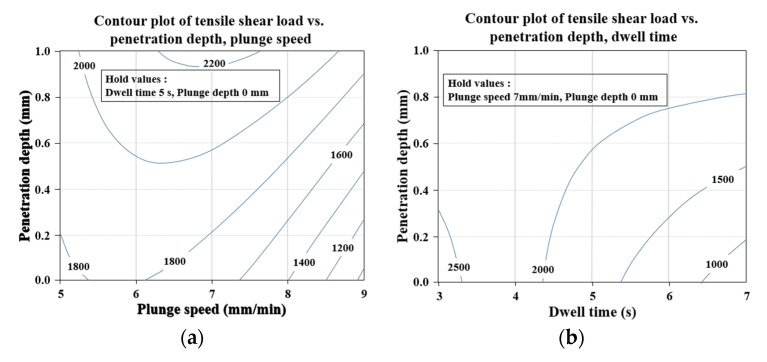

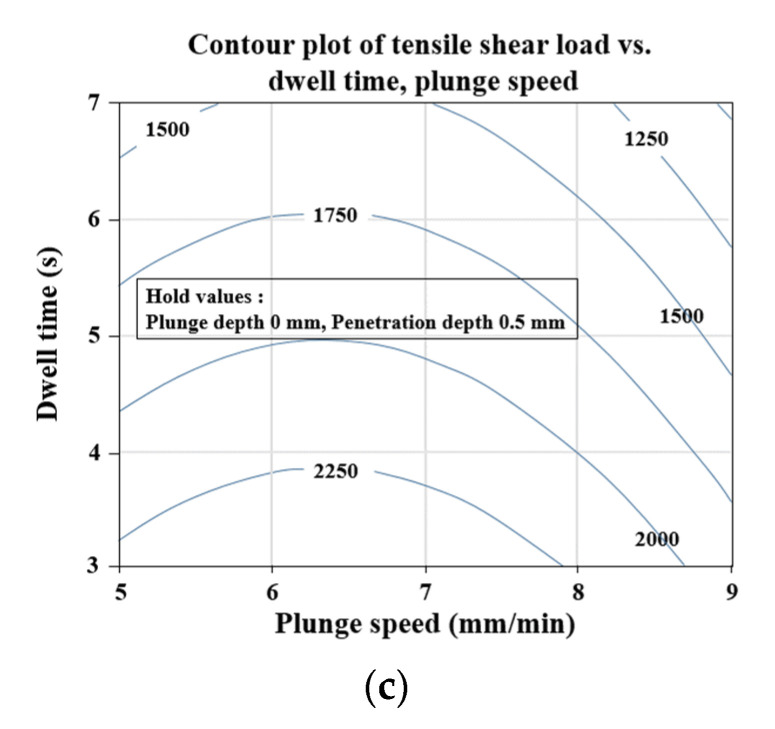
Contour plots of tensile shear load. (**a**) Penetration depth and plunge speed; (**b**) penetration depth and dwell time; (**c**) dwell time and plunge speed.

The plot also shows that the interaction between dwell time and tool penetration depth (D × F) is relatively low for plunge speed and tool penetration depth (C × F) without intersection. Figure 5 shows a contour plot for TSL, and in order to examine the effect of a given factor, fixed values of the remaining factors were selected by considering the main effects plot of Figure 3. Figure 5a shows the plane contour plot of the plunge speed and the tool penetration depth for TSL.

A dwell time of 5 s and a plunge depth of 0 mm were chosen for the fixed values of the remaining factors. On the contour plot, you can find the dwell time and tool penetration depth that maximize the TSL. Over the entire range of a given plunge speed, TSL increases with increasing tool penetration depth. Over the entire range of a given tool penetration depth, TSL increases with increasing plunge speed and decreases after reaching a maximum value. The high TSL value at low plunge speed and high tool penetration can also be seen in the main effect plot in Figure 3. Figure 5b shows the plane contour plot of the dwell time and the tool penetration depth for TSL. A plunge speed of 7 mm/min and a plunge depth of 0 mm were chosen from the main effects plots in Figure 3 as fixed values for the remaining factors. On the contour plot, you can find the dwell time and tool penetration depth that maximizes TSL. Over the entire range of a given tool penetration depth, increasing the dwell time decreases the TSL; for dwell times of less than 4 s in a given range, the TSL decreases with increasing tool penetration depth. For dwell times greater than 4 s in a given range, TSL increases with increasing tool penetration depth. It can be seen that in a given range, a maximum TSL value of about 2600 N appears at a dwell time of 3 s and a tool penetration depth of 0 mm. Figure 5c shows a plane contour plot of plunge speed and dwell time for TSL. A tool penetration depth of 0.5 mm and a plunge depth of 0 mm were chosen from the main effects plots in Figure 3 as fixed values for the remaining factors. On the surface plot, you can find the dwell time and plunge speed that maximize the TSL. Over the entire range of a given plunge speed, increasing the dwell time decreases the TSL. Over the entire range of a given dwell time, as the plunge speed increases, the TSL increases, reaching a maximum value and decreasing again after reaching the maximum value. The high TSL value at low dwell time and maximum plunge speed can also be seen from the main effect plots in Figure 3. It can be seen that a maximum TSL of about 2450 N in a given range appears at a dwell time of 3 s and a plunge speed of 6.3 mm/min.

**Figure 6:**
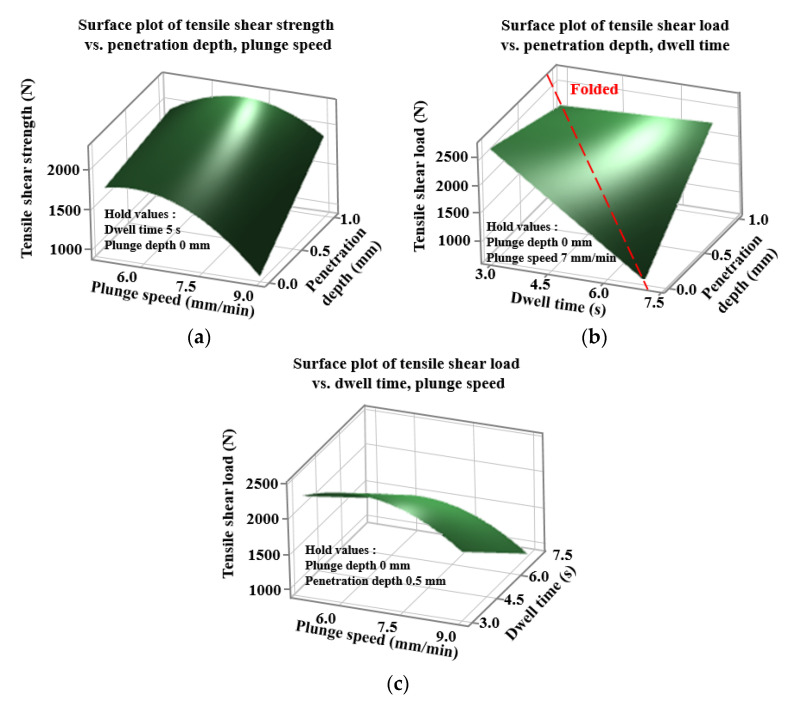
Surface plots of tensile shear load. (**a**) Penetration depth and plunge speed; (**b**) penetration depth and dwell time; (**c**) dwell time and plunge speed.

Figure 6 shows a surface plot for TSL. In order to examine the effect of a given factor, the fixed values of the remaining factors were selected in consideration of the main effect plot in Figure 3. Figure 6a shows a three-dimensional surface plot of plunge speed and tool penetration depth for TSL. A dwell time of 5 s and a plunge depth of 0 mm were chosen for the fixed values of the remaining factors. On the surface plot, you can find the plunge speed and tool penetration depth that maximize TSL. Over the entire range of a given plunge speed, TSL increases with increasing tool penetration depth. It can be seen that at a tool penetration depth of 0 mm, TSL increases with increasing plunge speed and decreases after reaching a maximum of about 1800 N. It can be seen that at a tool penetration depth of 0.5 mm, the TSL increases with increasing plunge speed and decreases after reaching a maximum value of about 2000 N. It can be seen that at a tool penetration depth of 1 mm, TSL increases with increasing plunge speed and decreases after reaching a maximum value of about 2200 N. A maximum TSL value of about 2200 N is achieved at a plunge speed of 7 mm/min and a tool penetration depth of 1.0 mm. Figure 6b shows a three-dimensional surface plot of dwell time and tool penetration depth for TSL. A plunge speed of 7 mm/min and a plunge depth of 0 mm were chosen from the main effects plots in Figure 3 as fixed values for the remaining factors. On the surface plot, you can find the dwell time and tool penetration depth that maximize the TSL. At a tool penetration depth of 0 mm, the TSL decreases with increasing dwell time. It can be seen that at a tool penetration depth of 0.5 mm, when the dwell time increases, the TSL decreases to the folding point and increases again after the folding point. At a tool penetration depth of 1 mm, the TSL increases with increasing dwell time. At a dwell time of 3 s, the TSL is decreasing with increasing tool penetration depth. At a dwell time of 5 s, as the tool penetration depth increases, the TSL decreases to the folding point and increases again after the folding point. It can be seen that at a dwell time of 7 s, increasing the tool penetration depth increases the TSL. It can be seen that in a given range, a maximum TSL value of about 2500 N appears at a dwell time of 3 s and a tool penetration depth of 0 mm. Figure 6c shows a three-dimensional surface plot of plunge speed and dwell time for TSL. A tool penetration depth of 0.5 mm and a plunge depth of 0 mm were chosen from the main effects plots in Figure 3 as fixed values for the remaining factors. On the surface plot, you can find the dwell time and plunge speed that maximize the TSL. Over the entire range of a given plunge speed, TSL decreases with increasing dwell time. Over the entire range of a given dwell time, as the plunge speed increases, the TSL increases, reaches a maximum value, and decreases again after reaching the maximum value. It can be seen that a maximum TSL of about 2250 N in a given range appears at a dwell time of 5 s and a plunge speed of 3 mm/min. In order to examine the effect of a given factor, the fixed values of the remaining factors were selected in consideration of the main effect plot in Figure 3.

The individual satisfaction function that maximizes the response$\;d_{i}$ is as follows.

(2) $$d_{i}=0,\;\;\;\;\hat{y_{i}}<L_{i}$$

(3) $$d_{i}={\left(\frac{\left(\hat{y_{i}}-L_{i}\right)}{\left(T_{i}-L_{i}\right)}\right)}^{r_{i}},\;\;\;L_{i}\leq \hat{y_{i}}\leq T_{i}$$

(4) $$d_{i}=1,\;\;\;\;\hat{y_{i}}>T_{i}$$

where $d_{i}$ is the individual desirability of the *i* th response; $\hat{y_{i}}$ is the expected response value of the *i* th response; $T_{i}$ is the target value of the *i* th response; $L_{i}$ is the minimum value of the *i* th response; $r_{i}$ is the weight of the desirability function of the *i* th response. The composite desirability function D is expressed as Equation (5).

(5) $$D={\left(\prod {\left(d_{i}\right)}^{w_{i}}\right)}^{\frac{1}{W}}\;\;\sum W_{i}=W$$

where $w_{i}$ is the importance of the *i* th response and *W* is $\sum W_{i}$.

**Figure 7:**
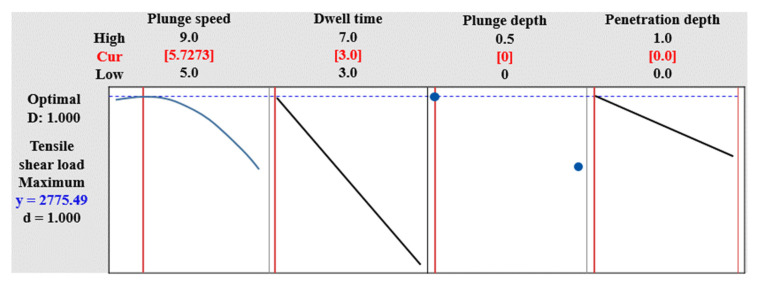
Response optimization of tensile shear load.

If there is one response variable and the importance is set to 1 as in Equation (5), individual desirability and composite desirability are the same. Figure 7 shows the reaction optimization for TSL. Among the factors in Figure 3, the tool rotation speed (A) and welding speed (B), which are factors that do not affect TSL, are not considered in the reaction optimization of Figure 7. Therefore, the response optimization value of TSL is found as the remaining four factors affecting TSL. The maximum and minimum levels in the experimental range for each factor are shown, and the maximum value of TSL is found within the combination of these four factors. Using the response optimization tool and the overall satisfaction function = 1 in Equation (5), the optimal conditions for factors maximizing TSL while satisfying the lower limit [2300 N] are shown. In the response optimization analysis, the optimal values of the derived response variables of four factors that satisfy the optimal conditions (mean TSL 2775.49 N, maximizing overall satisfaction (1.0)) are shown. The optimum value of Cur for each factor maximizing TSL is at a plunge speed of 5.7273 mm/min, a dwell time of 3.0 s, a plunge depth of 0 mm, and a tool penetration depth of 0 mm. This value lies between the maximum and minimum values of each factor level. It can be seen that the closer to the maximum value of the plunge speed, the shorter the dwell time, the lower the plunge depth, and the deeper the tool penetration depth, the closer the composite satisfaction is to 1 and the higher the TSL.

### 3.4. Microstructure Characteristics of Friction Stir Welding Joint

**Figure 8:**
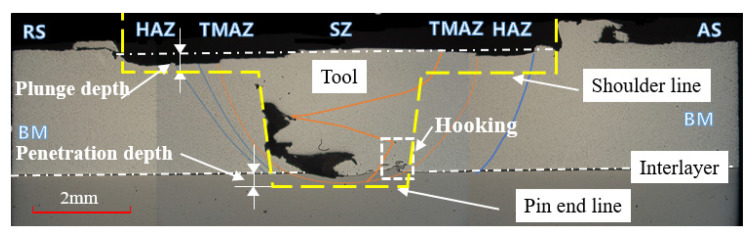
Optical microscope image of the cross-section of lap joints and the dimensions of plunge depth and tool penetration depth.

Figure 8 illustrates a magnified view of the microstructure photograph of the FSLW of the A357 cast Al and FB590 high-strength steel pipes and the region of interest around the interface of the dissimilar material to observe the effects of the plunge depth and tool penetration depth.

The shape of the tool pin is indicated by a thick dotted line, and the interlayer is indicated by a dashed-dotted line where A390 Al and FB590 high-strength steel are in contact.

The plunge depth represents the arrow gap between the outer diameter of the A390 Al pipe and the tool shoulder, and the tool penetration depth represents the arrow gap between the boundary layer and the end line of the tool pin. After FSW, there is a stir zone (SZ) around the center of the joint, a thermo-mechanically affected zone (TMAZ) in which grains are increased by the plastic flow on the outside of the SZ, and a heat-affected zone (HAZ) that is heat-affected but has no plastic deformation on the outside of the TMAZ. These zones were observed to have a wider area width than the RS in the tool’s AS. In the hooking area, it can be observed that some particles of the steel are raised toward aluminum in a hook shape, indicating that the two materials are physically bonded, and steel fractures are visible around the hooking area. Most of the steel fragments adhered near to AS of the tool pin. In addition, it was observed that steel particles were deposited away from the interface along AS, whereas they were near the interface along RS [40]. Surface defects such as weld flashes and surface grooves were observed at different plunge lengths and tool penetration depths under experimental conditions. The volume of the welding flash increased further as the welding progressed. Similar results were observed by Das et al. [56]; that is, the penetration depth of the tool pin into the workpiece (also known as the target depth) is important for producing a sound weld. They reported that if the plunge depth of the tool is too shallow, the shoulder of the tool will not touch the original workpiece surface, creating a weld with internal channels or surface grooves, and if the plunge depth of the tool is too deep, the shoulder of the tool will enter the workpiece, and cause excessive flash. In contrast, excessive flash and internal cavities were found with and without plunge depth in this study.

**Figure 9:**
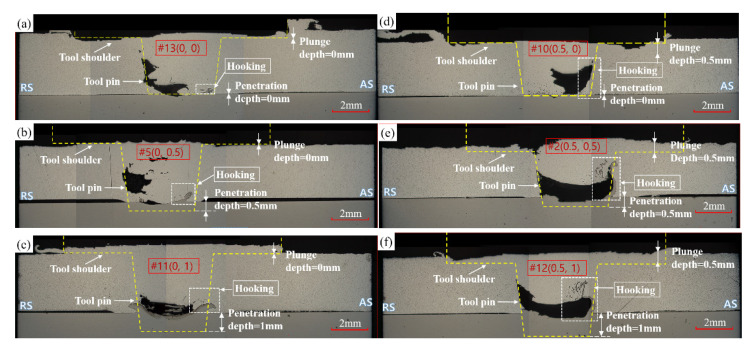
Comparison of the plunge depth and penetration depth on the interface structure. (**a**) (0 mm, 0 mm) (test No. 13); (**b**) (0 mm, 0.5 mm) (test No. 5); (**c**) (0 mm, 1 mm) (test No. 11); (**d**) (0.5 mm, 0 mm) (test No. 10); (**e**) (0.5 mm, 0.5 mm)(test No. 2) (**f**) (0.5 mm, 1 mm) (test No. 12).

Figure 9 shows the microstructure of the specimen after the friction stir test according to the order determined by the corresponding test No. 1–14 for plunge depth and tool penetration depth (a) 0 mm, 0 mm; (b) 0 mm, 0.5 mm; (c) 0 mm, 1 mm; (d) 0.5 mm, 0 mm; (e) 0.5 mm, 0.5 mm; and (f) 0.5 mm, 1 mm, respectively. The picture was taken in the direction of 90 degrees perpendicular to the progress direction of the welding part. Internal welding defects occurred in most of the experimental areas. Major internal defects occur in the form of wormholes, cavities, tunnel defects, and voids and are caused by the lack of material to fill the cavity formed by the flash in the weld area. The cause of the internal cavity is that the region where excessive heat is generated was selected in the process of finding a region where external defects do not occur in different tool pin lengths. The reason for choosing this experimental area is that it is reasonable to select it based on the experimental area of the tool speed and welding speed of Choy et al. [37], where there were no external defects using a tool with fixed single pin-length. In the process of selecting the experimental area with few external defects around this area, the area with similar tool rotation speed and low welding speed was selected.

As a result, the FSW experiment was conducted in an area where excessive heat was generated. The high heat generated in the weld excessively softens the material inside the weld, and the plasticized material (caused by the continuous stirring action of the tool) is expelled out of the weld in the form of a flash. This leads to the loss of material for completely filling the cavity formed by the rotating tool and results in voids inside the weld. As the process is continuous, the voids extend along the length of the weld to form a tunnel defect. The flow of plasticized material in the FSW process occurs from the advancing side to the RS toward the trailing edge of the tool. The heat and constant rotation of the tool forge the plasticized material toward RS and deposits it in the RS, filling the cavity formed around the tool. Consistent with Arbegast’s [57] results, internal weld defects due to insufficient material for the trailing edge of the tool because of over-softening under high-temperature conditions are observed. TSL is affected by plunge depth and tool penetration depth. It was found that as the plunge depth decreased and the tool penetration depth increased, the TSL value increased. On the other hand, according to the geometrical characteristics of FSLW, in experiments No. 13, 5, and 11, a hooking part could be found on the boundary layer, and it was found that the size of the hooking part was affected by the tool penetration depth. Experiment No. 2 and 12 show that, owing to the size and location of the cavity, the hooking part was formed following the shape of the cavity from the boundary layer. However, in experiment No. 10, the hooking part did not form. This seems to be the effect of the difference in the plunge depth when compared with No. 13, which had a penetration depth of 0 mm. In No. 13, where the plunge depth is 0 mm, there is no flash effect by the plunge depth; hence, even if there is no penetration depth, hooking occurs in the process of forming the joint by transmitting the rotational force of the tool. In the case of No. 10, with a plunge depth of 0.5 mm, the material for the hooking part disappears into the cavity, owing to the lack of material to fill the cavity due to the flash effect by the plunge depth; hence, the effect of hooking does not appear. It can be seen that the formation position of the cavity tends to form stably from a one-way bias away from the center of the tool as the tool penetration depth increases and the size of the cavity decreases.

## 4. Conclusions

FSLW with pipe-type A357 cast Al and FB590 high-strength steel was investigated. Optimized tools were used, and the tool rotational speed, tool welding speed, plunge speed, dwell time, plunge depth, and tool penetration depth were selected among the process variables; and the response was studied through the TSL values. After conducting a total of 14 experiments with 6 factors and 3 levels (excepted for plunge depth, which had 2 levels), the following major results were obtained through tensile test measurements:

(1) Using DSD techniques, which allow for simultaneous identification of the effects of multiple factors with low cost and time, we managed to identify the effects of various process factors in addition to rotational and weld speeds—which are known as the most important factors in FSW—and identified the factors affecting TSL.

(2) Among the process factors selected for the friction stir welding of pipe-type Al and steel, the impact of flange depth and tool penetration depth was most significant to TSL, and an independent relationship with no interaction between plunge depth and tool penetration depth was identified. The plunge depth has a negative effect, and the tool penetration depth has a positive effect on the magnitude of the TSL.

(3) The weak interaction effect between the plunge speed and tool penetration depth and the strong interaction effect between the dwell time and tool penetration depth were confirmed. The folding phenomenon of the interaction between tool penetration depth and dwell time was found to have an opposite effect on TSL depending on the direction of increase or decrease of the factor.

(4) The depth of tool penetration and plunge depth affected the cavity size; the tool penetration depth contributed to the stabilization and size reduction of the cavity.

(5) It was found that the tool penetration depth had the greatest influence on the size of the hooking part in the lap welding of the pipe; moreover, the hooking part, which was not created on the boundary layer by the increase in the plunge depth, was distributed in a certain size along the shape of the cavity.

By confirming the influence of process factors through DSD experiments, we reached the important conclusion that when selecting a tool, the plunge depth and tool penetration depth should be considered, especially in FSLW. This will play an important role in future dissimilar FSLW experiments and can be utilized for optimization through the additional selection of levels.

## Figures and Tables

**Table 1:** Chemical composition of FB590 (high-strength steel) and A357 (cast Al) pipes [9].

Material	C	Si	Mn	P	S	Cr	Ni
FB590	0.076	0.094	1.472	0.013	0.001	0.019	0.008
Material	Si	Mg	Cu	Zn	Fe	Mn	Ti
A357	6.937	0.507	0.034	0.017	0.181	0.007	0.116

**Table 2:** Process parameters (factors) and their levels.

	Factors	Tool Speed (rpm)	Welding Speed (rpm)	Plunge Speed (mm/min)	Dwell Time (s)	Plunge Depth (mm)	Penetration Depth (mm)
Level							
−		1700	0.10	5	3	0	0
0		1800	0.15	7	5	-	0.5
+		1900	0.20	9	7	0.5	1

**Table 3:** Experiment level with run order.

No.	Tool Speed (rpm)	Welding Speed (rpm)	Plunge Speed (mm/min)	Dwell Time (s)	Plunge Depth (mm)	Penetration Depth (mm)	Tensile Shear Load (N)
Symbol	A	B	C	D	E	F	TSL
1	1900	0.2	5	3	0.5	0.5	1440.28
2	1800	0.15	7	5	0.5	0.5	1414.87
3	1800	0.2	9	7	0.5	1	1112.12
4	1900	0.2	7	7	0	0	807.35
5	1700	0.1	9	7	0	0.5	860.50
6	1800	0.15	5	5	0	0.5	1779.25
7	1900	0.15	5	3	0	1	2000.99
8	1700	0.15	7	7	0.5	0	0
9	1700	0.2	7	3	0.5	0	1238.16
10	1900	0.1	5	5	0.5	0	0
11	1900	0.1	5	7	0	1	2034.85
12	1900	0.1	7	3	0.5	1	1182.08
13	1800	0.1	5	3	0	0	2677.21
14	1700	0.2	5	5	0	1	1984.72

**Table 4:** Model summary.

S	R-sq	R-sq (adj)	R-sq (pred)
197.261	96.90%	93.27%	81.18%

**Table 5:** Analysis of variance.

Source	DF	Adj SS	Adj MS	F-Value	P-Value
Model	7	7,287,025	1,041,004	26.75	0.000
Linear	4	5,644,173	1,411,043	36.26	0.000
Plunge speed	1	432,946	432,946	11.13	0.016
Dwell time	1	1,995,350	1,995,350	51.28	0.000
Plunge depth	1	2,111,423	2,111,423	54.26	0.000
Penetration depth	1	749,284	749,284	19.26	0.005
Square	1	237,734	237,734	6.11	0.048
Plunge speed x Plunge speed	1	237,734	237,734	6.11	0.048
Two-way interactions	2	1,638,507	819,253	21.05	0.002
Plunge speed x Penetration depth	1	280,384	280,384	7.21	0.036
Dwell time x Penetration depth	1	1,603,050	1,603,050	41.20	0.001
Error	6	233,471	38,912		
Total	13	7,520,496			

## Data Availability

Not Applicable.
